# Congenital Melanocytic Macule of the Tongue in a Newborn

**DOI:** 10.7759/cureus.11475

**Published:** 2020-11-13

**Authors:** Lilian Ablan, Catalina Acosta, Alexander Rodriguez

**Affiliations:** 1 Pediatrics, Woodhull Medical Center, Brooklyn, USA; 2 Neonatalogy, Woodhull Medical Center, Brooklyn, USA

**Keywords:** congenital melanocytic macule of the tongue (cmmt), newborn, asymptomatic, macule.

## Abstract

We report the first case of a Hispanic infant with a congenital hyperpigmented macule of the tongue at Woodhull Medical Center in Brooklyn, NY.

## Introduction

Congenital melanocytic macule of the tongue (CMMT) is a rare entity with a benign course usually reported in dark-skinned and Caucasian patients [1]. Our literature review only showed a limited number of cases in Hispanic individuals. Previous authors have reported CMMTs as multiple, asymptomatic, well-defined homogeneous- or heterogenous-colored lesions on the dorsum of the tongue [1-3].

This case report would aim to increase awareness about this benign skin condition among the general pediatricians practising in nursery and neonatal intensive care unit (NICU) setting. Because there are technical difficulties associated with invasive procedures, such as surgery or biopsy, that are performed on a child, close follow-up of this type of lesions should be performed. The lesions would likely increase in size with a child’s growth [2]. We report the first case of a Hispanic infant with a congenital hyperpigmented macule of the tongue at Woodhull Medical Center in Brooklyn, New York, USA.

## Case presentation

**Figure 1:**
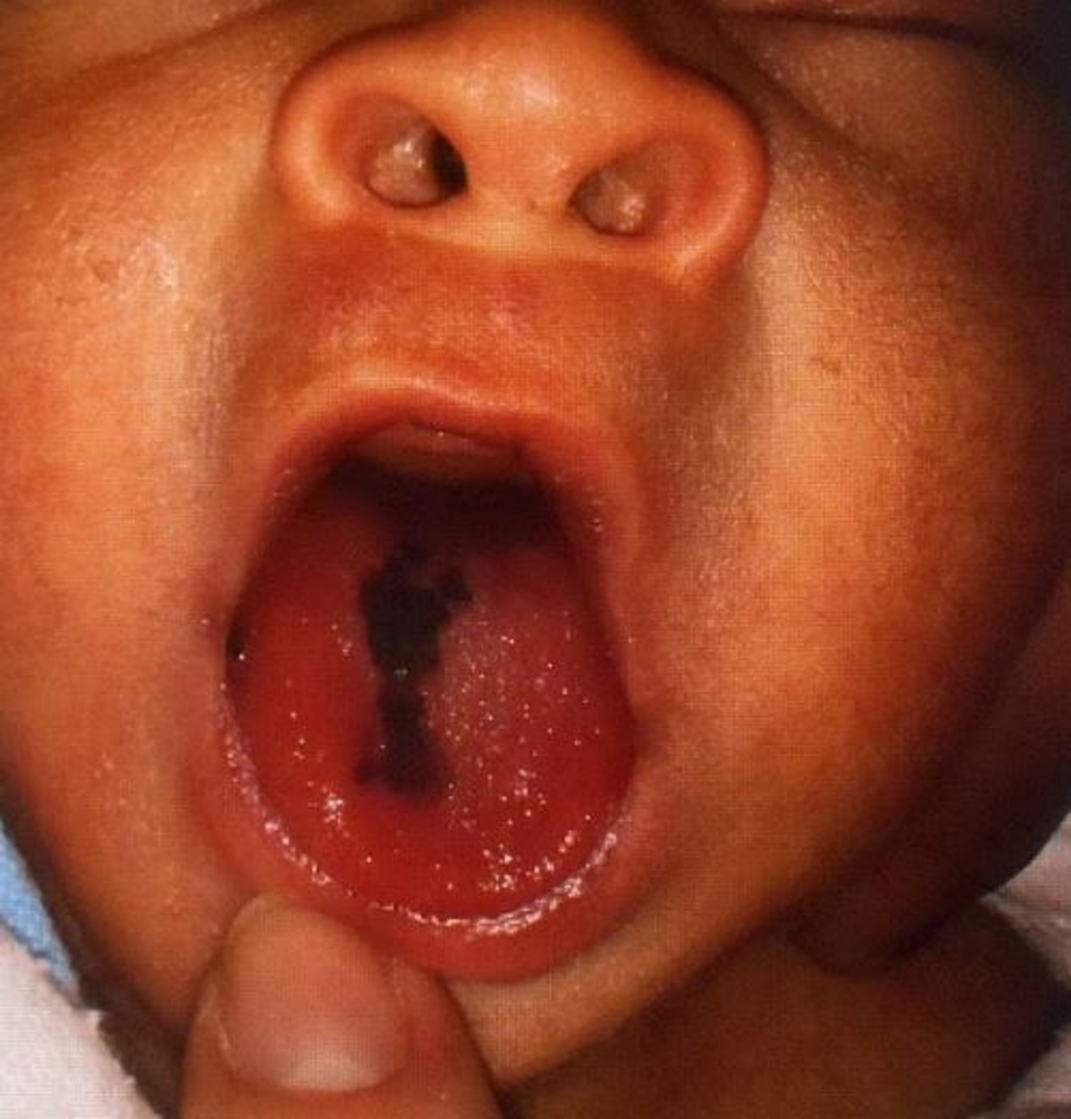
Single homogeneous pigmented lesion, 5 x 15 mm, irregular, dark brown, on the right dorsum of the tongue

A full-term male newborn (39 weeks) of Hispanic origin presented with a pigmented lesion on the tongue, which was noted at birth. A physical exam showed an active newborn, skin phototype IV, and a single 5x15 mm, irregular, dark-brown, homogeneous pigmented lesion on the right dorsum of the tongue (Figure 1); the remaining findings of the examination were within reference limits.

Regarding his birth history, the infant was a product of an uneventful pregnancy, born via Caesarean section due to breech presentation. His family history is negative for melanoma, polyposis, mucosal pigmentation, von Recklinghausen’s disease, and Addison’s disease, as well as other skin disorders.

**Figure 2:**
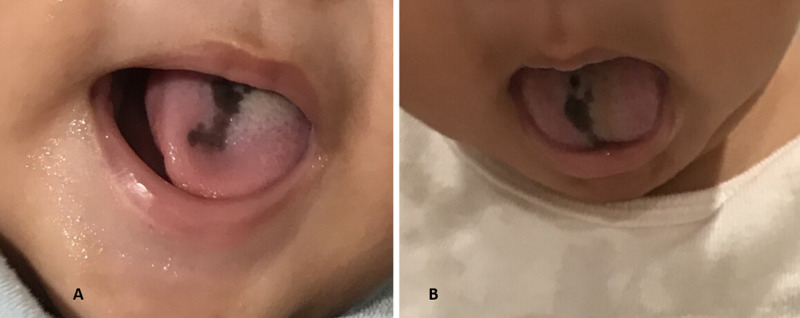
A) Single homogeneous pigmented lesion shown at seven months after birth. B) Second view of the lesion.

In this case, CMMT was diagnosed in the absence of concomitant systemic diseases and following staff review of clinical findings, location on the lesion, negative family history for melanoma, and the absence of drugs and toxic exposure during gestation. Rather than undergoing a tongue biopsy, which is challenging to perform on an infant, the patient underwent focused clinical monitoring by the dermatology department every three months, for the next 30 consecutive months. The patient received no medications, and at seven months of age, the patient remained asymptomatic, and the macule was slightly faded, with an increase in size that was relative to the child’s normal growth (Figure 2A, 2B).

## Discussion

CMMT in the newborn is a benign and rare entity first described in the early 2000s [1,2]. These hyperpigmented lesions can be single or multiple, asymptomatic, with diameters that vary from 0.3 to 3 cm, and with benign histological characteristics [2,4].

The prevalence of oral lesions in children is not well established. In a cross-sectional study of 1,211 Brazilian children, the authors reported a 27% prevalence for oral alterations such as geographic tongue, cheek biting, and melanotic macule [5]. Interestingly, the authors also mentioned that mucosal alterations’ frequency increases with age, habits, and medical history. Furthermore, geographic tongue, pseudomembranous candidiasis, and alveolar cyst were more frequent in children up to four years old, while melanotic macule, fissured tongue, and recurrent herpes were more prevalent in older children.

Oral hyperpigmentation seen in adults can be related to smoking, radiation, drugs, amalgam tattoo, melanoma, and Peutz-Jeghers syndrome, among others. For accurate diagnosis and management, a biopsy, history, and physical exam are necessary [6].

In a case described by Savoia et al. of a 10-week-old infant, they did not perform a tongue biopsy, but instead, they followed the patient every three months up to 30 months and noticed no changes in the lesion, which indicated its benign nature [2]. Clinical follow-up may be sufficient to monitor CMMT rather than performing a tongue biopsy because the difficulty is increased when this procedure is performed on a newborn.

In some cases, biopsies were performed, and the histologic features are an excess deposition of melanin in the basal cell layer and lamina propria, with a normal number of melanocytes and no melanocyte nests (which are characteristics that are different from melanomas). Additionally, there is an increase in the basal epidermal layer’s melanin pigmentation and hyperkeratosis associated with abundant subepidermal pigment-laden macrophages [4].

Limitations

This report’s limitations include the paucity of data regarding CMMT in the literature, which led to a brief review of the treatment algorithm. We suspect that the reason for this scarcity could be the low rate of reports of existing cases.

## Conclusions

Congenital melanocytic macule of the tongue is a rare but benign entity with an underestimated prevalence. General pediatricians usually have low exposure to this condition resulting in failure to recognize and underdiagnosis. In most cases, frequent follow-ups are conducted to ensure that the lesion is not presenting with acute changes in shape or color that might suggest a different diagnosis. To date, there is no specific treatment for this condition. In this case, the patient received no medications, and at seven months of age, remained asymptomatic. However, even though the patient was monitored every three months, a biopsy was indicated.
